# Peri-implant mucositis and peri-implantitis: key features and differences

**DOI:** 10.1038/s41415-024-7402-z

**Published:** 2024-05-24

**Authors:** Lisa J. A. Heitz-Mayfield

**Affiliations:** The University of Western Australia, International Research Collaborative, Oral Health and Equity, School of Human Anatomy and Biology, Crawley, WA, Australia; The University of Sydney, School of Dentistry, Faculty of Medicine and Health, NSW, Australia

## Abstract

Peri-implant diseases are frequent complications that occur around osseointegrated endosseous implants and are the result of an imbalance between the bacterial challenge and host response. Peri-implant diseases may affect the peri-implant mucosa only (peri-implant mucositis) or also involve the supporting bone (peri-implantitis). Early detection of peri-implant diseases and timely treatment is important for the success of dental implant treatment. Peri-implant probing is essential to assess the peri-implant health status and should be done at each recall visit. Dental practitioners should be familiar with the clinical and radiological features of both conditions in order to make an accurate diagnosis and determine the appropriate treatment required. This article aims to provide clinicians with an understanding of the key differences between peri-implant health, peri-implant mucositis and peri-implantitis.

## Key points

- Peri-implant mucositis is characterised by clinical signs of inflammation (bleeding on probing) without loss of supporting bone.
- Peri-implantitis is characterised by clinical signs of inflammation (bleeding on probing) in addition to progressive bone loss.
- Peri-implant probing is essential for the diagnosis of the peri-implant health status and should be performed at each recall visit.

## Introduction

Peri-implant diseases, peri-implant mucositis and peri-implantitis refer to biofilm-associated inflammatory conditions that affect the peri-implant tissues. Both peri-implant mucositis and peri-implantitis are frequent complications following implant treatment^1^ and it is important for clinicians to be able to make a correct diagnosis in order to make appropriate treatment decisions.

Peri-implant mucositis is considered the precursor to peri-implantitis, a condition which may progress rapidly, leading to advanced bone loss, resulting in loss of an implant.

Early detection of peri-implant disease and early intervention is key to prevent disease progression, preserve implant longevity and ensure patients' overall satisfaction and quality of life. In this narrative review, the key clinical, radiologic and histologic features of peri-implant mucositis and peri-implantitis are presented, highlighting the differences between the two inflammatory conditions.

## Clinical signs of peri-implant health

Healthy peri-implant soft tissues (termed peri-implant mucosa) can be achieved when an implant is correctly placed (ie in an appropriate three-dimensional position surrounded by an adequate volume of bone) and restored with a well-designed and fabricated prosthesis in a patient with good plaque control and oral health. Following the completion of oral rehabilitation involving dental implants, the clinician should measure and record circumferential peri-implant probing depths and soft tissue levels (four to six sites) to establish a baseline for future comparisons. It is also recommended to assess and record the width of keratinised peri-implant mucosa surrounding the implant.

**Fig. 1:**
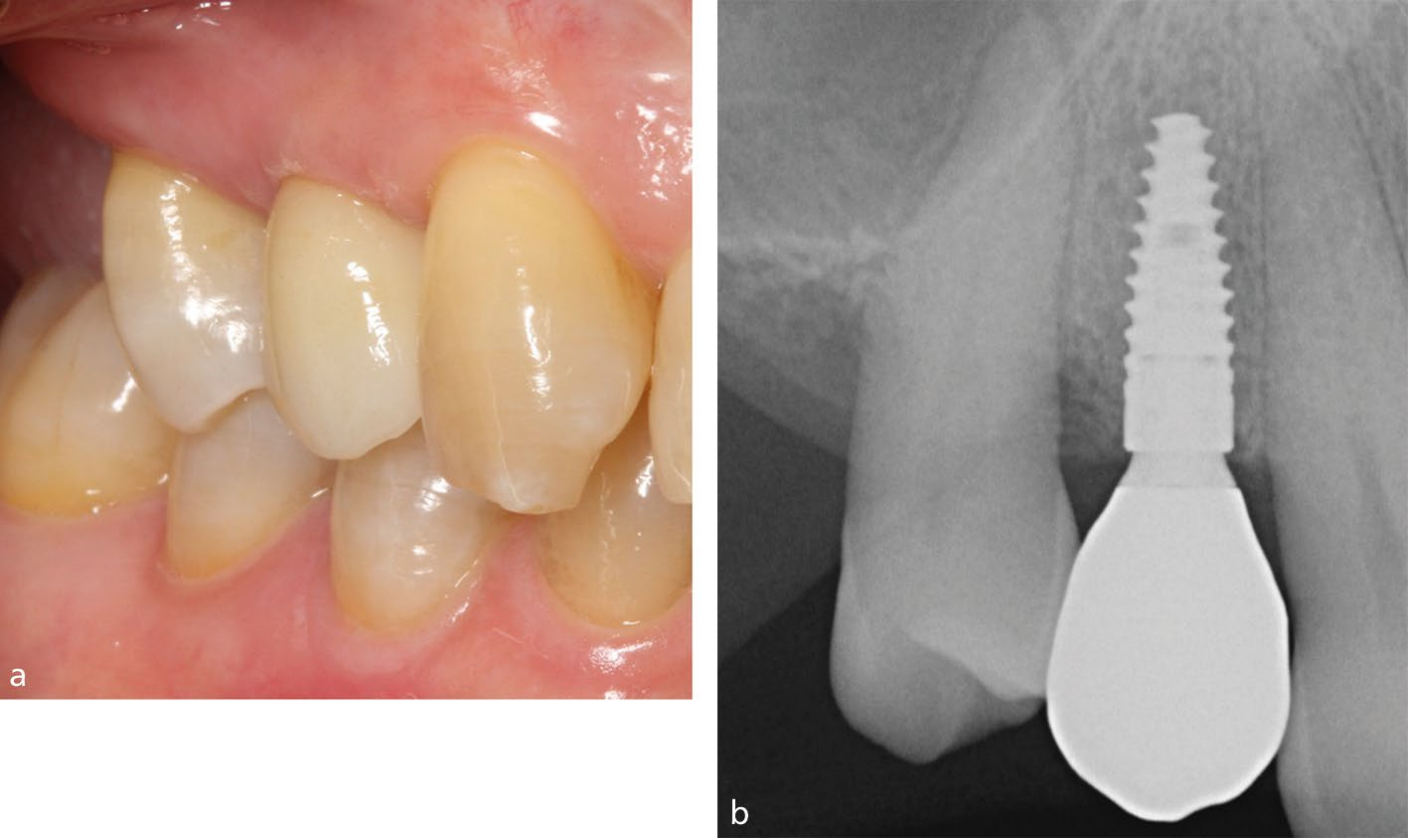
a) Healthy peri-implant mucosa at the implant-supported crown in the upper right first premolar site. b) Periapical radiograph showing marginal bone levels after remodelling with no loss of supporting bone

Healthy peri-implant mucosa should have a similar appearance as that of healthy gingiva (Fig. 1a). There should be no visual signs of inflammation such as erythema (redness) or oedema (swelling) and when the peri-implant sulcus is probed circumferentially (at four to six sites) using a periodontal probe with a light probing force (approximately 0.2 N), there should be no bleeding. Histologic features of healthy peri-implant tissues are described in the paper by Araujo in this themed issue.^2^

## Radiologic signs of peri-implant health

After implant placement and during the healing period, physiologic bone remodelling occurs and the peri-implant marginal bone levels are established at, or slightly below, the most coronal portion of the endosseous part of the implant. Once an implant is restored, an intra-oral radiograph (periapical or bitewing) should be made to identify the peri-implant bone levels at the mesial and distal aspect of the implant. This establishes baseline marginal bone levels in health and serves as a reference for monitoring changes in marginal bone levels over time (Fig. 1b).

## Clinical and radiologic signs of peri-implant mucositis

**Fig. 2:**
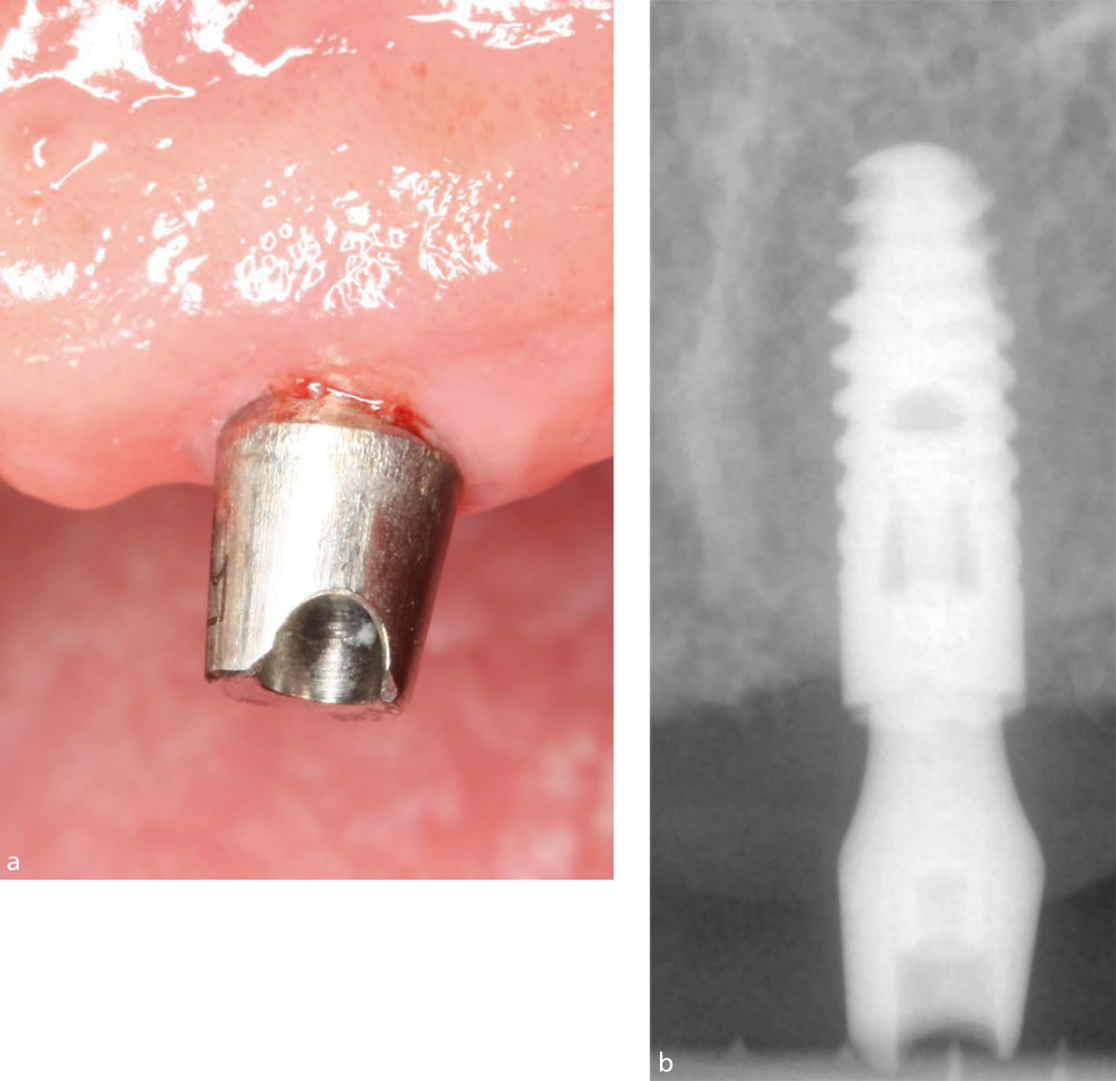
a) Peri-implant mucositis showing inflammation and bleeding on light probing. b) Periapical radiograph showing no loss of supporting bone

The main criteria for the definition of peri-implant mucositis are inflammation in the peri-implant mucosa and the absence of continuing marginal peri-implant bone loss.^3^ The main clinical sign of inflammation is bleeding following gentle probing (0.2 N), while additional signs may include redness, swelling and suppuration (pus). When peri-implant mucositis is present, there may also be deepening of the peri-implant probing depths compared to the baseline probing measurements made following delivery of the implant prosthesis. When these clinical signs are detected during examination, a radiograph demonstrating the absence of marginal bone loss confirms the diagnosis of peri-implant mucositis (Fig. 2a and b).

It should be noted that, according to the European Federation of Periodontology S3 clinical practice guideline on the treatment of peri-implant diseases, published in 2023, the presence of bleeding on probing (BoP) refers to more than one spot at a location around the implant, or the presence of a line of bleeding or profuse bleeding at any location.^4^

## Histologic features of peri-implant mucositis

Peri-implant mucositis occurs following accumulation of microbial biofilms around dental implants. Experimental human studies allowing accumulation of bacterial biofilms for a period of three weeks have shown a cause-and-effect relationship between bacterial biofilms and the development of an inflammatory response.^5^^,^^6^^,^^7^^,^^8^ In one study, biopsies of peri-implant tissues after 21 days of plaque accumulation showed that the connective tissue surrounding the implants was characterised by an greater volume of inflammatory cells (T- and B-lymphocytes) compared to biopsies from healthy peri-implant tissue.^7^ Biopsies obtained from the peri-implant mucosa around implants in humans with long-standing peri-implant mucositis have found small, well-defined inflammatory infiltrates in the connective tissue lateral to the barrier epithelium of the peri-implant pocket^9^ and an increase in the size of the inflammatory lesion compared to clinically health sites.^10^

Peri-implant mucositis is considered a reversible condition and experimental studies in humans have shown that, following biofilm removal, there is a reversal of the elevation of inflammatory biomarkers measured in the peri-implant sulcular fluid.^6^^,^^8^ However, complete resolution of the clinical signs of inflammation may be more difficult to achieve, as shown in an experimental peri-implant mucositis study, when biofilm control for three weeks did not result in complete resolution at a clinical level.^6^ Another experimental peri-implant mucositis study showed that when implants are deeply placed with deep submarginal prosthetic margins creating a deep mucosal sulcus, resolution is difficult to achieve.^11^

As peri-implant mucositis is considered a precursor to peri-implantitis and a risk factor for peri-implantitis, clinicians are advised to promptly treat peri-implant mucositis and re-evaluate the outcome of treatment as a preventative measure against the progression of peri-implant mucositis to peri-implantitis.

## Clinical and radiologic signs of peri-implantitis

Peri-implantitis is a pathological condition which occurs in the tissues surrounding dental implants, characterised by inflammation in the peri-implant connective tissue and progressive loss of supporting bone.^12^

**Fig. 3:**
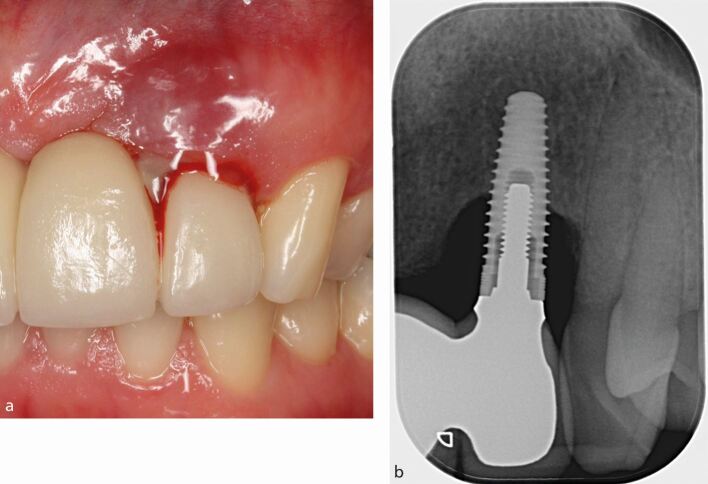
a) Peri-implantitis at the implant in the upper lateral incisor site, with deep peri-implant probing depths >6 mm, redness, swelling, bleeding and suppuration on probing. b) Periapical radiograph showing marginal bone loss >3 mm apical to the most coronal part of the endosseous portion of the implant

Peri-implant probing to identify the presence of BoP is essential for the diagnosis of peri-implantitis. Sites with peri-implantitis have clinical signs of inflammation (BoP), increased probing depths and/or peri-implant mucosal recession compared to baseline recordings. Visual inspection of the implant site may identify additional clinical signs of inflammation including redness, swelling, or the presence of a draining sinus. Palpation of the peri-implant site may also identify suppuration from the peri-implant pocket (Fig. 3a and b).

An intra-oral radiograph to identify progressive marginal bone loss is required to confirm a diagnosis of peri-implantitis. When previous radiographs are not available for comparison, marginal bone levels apical to what would be expected following physiologic remodelling may be used to estimate the amount of loss of supporting bone.

In the absence of previous examination data (radiographs, probing measurements), a diagnosis of peri-implantitis can be made based on the combination of the presence of BoP, probing depths ≥6 mm and a marginal bone level ≥3 mm apical to the most coronal portion of the endosseous part of the implant.^13^

Peri-implant bone loss is usually circumferential and progression may occur early (within the first three years of function) and in a non-linear fashion.^14^

Detecting incipient (early) peri-implantitis requires evaluation of well-standardised intra-oral radiographs. This requires the use of a standardised film/sensor holder and a paralleling technique in order to minimise angulation errors and accurately detect changes in marginal bone levels. A threshold of 0.5 mm is used for the detection of bone level change.^13^ While intra-oral radiographs have the limitation of not providing visualisation of the buccal and oral peri-implant bone levels, they are considered the standard of care for assessment of peri-implant bone levels. Three-dimensional radiography, such as cone beam computed tomography (CBCT), may provide information regarding the facial and oral bone levels but CBCT scans are not recommended as a routine evaluation method.

## Histologic features of peri-implantitis and comparison to peri-implant mucositis

Characteristics of peri-implantitis lesions have been described following biopsies of peri-implantitis lesions from humans.^15^ Soft tissue biopsies obtained from implants with advanced bone loss and clinical signs of severe inflammation, including suppuration and swelling, showed the presence of large proportions of B-cells, neutrophils and macrophages.^9^ When compared to peri-implant mucositis lesions, the peri-implantitis lesions were found to be considerably larger in size and contained greater proportions of B-cells, as well as a higher density of vascular structures outside and lateral to the cell infiltrate.^9^ A further study in humans showed that cytokines with a potential to stimulate the formation and activity of osteoclasts (IL1-alpha, TNF-alpha, IL-6) were associated with peri-implantitis lesions.^16^

Animal studies, designed to evaluate the progression of experimental, ligature-induced peri-implantitis, have shown that the inflammatory cell infiltrate may be found in close proximity to the bone marrow spaces. Hence, the lesion may progress without the presence of a healthy connective tissue fibre compartment walling off the lesion from the alveolar bone.^17^^,^^18^ This is in contrast to the periodontitis lesion at teeth where the inflammatory lesion is separated by an intact, supracrestal connective tissue fibre compartment.^19^

From a clinical perspective, this means that, in some patients, peri-implantitis lesions may progress rapidly, emphasising the importance of early diagnosis, timely treatment and regular follow-up to evaluate treatment outcomes and provide appropriate supportive peri-implant care.

## Conclusion

- Peri-implant mucositis affects the peri-implant mucosa (soft tissues) and is characterised by clinical signs of inflammation (BoP) without loss of supporting bone
- Peri-implantitis is characterised by clinical signs of inflammation (BoP) in addition to progressive bone loss
- While not all peri-implant mucositis lesions progress to peri-implantitis, peri-implant mucositis is considered a precursor and risk factor for peri-implantitis
- The onset of peri-implantitis may occur early (within the first three years) and progression may be rapid. Peri-implantitis lesions differ from peri-implant mucositis lesions in that they extend beyond the pocket epithelium, have a larger size of inflammatory cell infiltrate and are characterised by large proportions of plasma cells
- It is recommended to obtain baseline radiographs and peri-implant probing measurements following completion of implant therapy. These recorded baseline data can serve as a reference for monitoring and detecting changes in marginal bone levels and probing depths over time, enabling early diagnosis of peri-implant diseases
- Peri-implant probing using a periodontal probe with light force (approximately 0.2 N) is essential for the diagnosis of the peri-implant health status and should be performed at each recall visit
- When clinical signs of inflammation are detected (BoP, deepening probing depths), an intra-oral radiograph confirms a diagnosis depending on whether there has been progressive or continuing peri-implant bone loss (peri-implantitis) or not (peri-implant mucositis).
